# Leukaemia incidence among workers in the shoe and boot manufacturing industry: a case-control study

**DOI:** 10.1186/1476-069X-3-7

**Published:** 2004-08-30

**Authors:** Steven P Forand

**Affiliations:** 1 Bureau of Environmental and Occupational Epidemiology, New York State Department of Health, 547 River Street: Room 200, Troy, New York 12180, USA

## Abstract

**Background:**

Previous reports have indicated an excess of leukaemia in Broome County, New York, particularly in the Town of Union. Surveillance of cancer incidence data indicates that a large proportion of these cases occurred among males ages 65 and older. Shoe and boot manufacturing has been the largest single industry in this area throughout much of the past century. Occupational studies from Europe suggest a link between leukaemia and employment in the shoe and boot manufacturing industry. However, researchers have not found a positive association between leukaemia and employment in the shoe industry among workers in the United States.

**Methods:**

A matched case-control study was conducted to investigate the association between leukaemia incidence among males 65 and older and employment in the shoe and boot manufacturing industry. Thirty-six cases of leukaemia occurring between 1981–1990; among males age 65 and older; residing in the town of Union met the study case criteria. Death certificates were obtained for each of the cases. These were matched to death certificates of 144 controls on date of death and date of birth +/- 1 year. Death certificates were then examined to determine the employer and occupation of each study subject. Conditional logistic regression was used to determine the risk of leukaemia among those working in the industry.

**Results:**

The risk of both leukaemia (OR = 1.47; 95% CI 0.70, 3.09) and acute myeloid leukaemia (OR = 1.19; 95% CI 0.33, 4.28) were elevated among those employed in the shoe and boot manufacturing industry, however neither was statistically significant.

**Conclusion:**

The results, though suggestive of an association between leukaemia and employment in the shoe and boot manufacturing industry, were not statistically conclusive due mainly to limited study power. Several additional limitations may also have prevented the observance of more conclusive findings. Better exposure assessment, information on length of exposure and types of job held, control of confounding factors and information on chemicals used by this company would strengthen any future investigation.

## Background

A number of previous reports have indicated an excess of leukaemia in Broome County, New York, particularly in the Town of Union [1-5]. The Broome County Health Department in conjunction with the New York State Department of Health (NYSDOH) conducted a study of cancer incidence among residents of several communities in Broome County for the years 1976–1980 and found a significant excess of leukaemia among males in the village of Endicott, which is located in the Town of Union [1]. In a follow up study, NYSDOH investigated cancer incidence for the years 1981–1990 [2]. As in the previous study, leukaemia was elevated among males in the Village of Endicott, although the findings were not statistically significant. When data were evaluated for the neighbouring village of Johnson City as well as for the entire Town of Union, significantly elevated rates of leukaemia were observed among males in both areas (unpublished data). When the data were examined more closely, it was noted that the majority of the leukaemia excess was limited to males ages 65 and older (Table 1). In addition, an atlas of cancer incidence in New York State between 1992 and 1996 shows that Broome County continues to have a statistically significant elevation of leukaemia among males [3].

**Table 1:** Leukaemia incidence by age among males in the Town of Union, Broome County, NY: 1981–90.

**Age Group**	**Number of cases**		**SIR**	**95% C. I.**^a^	
	Observed	Expected^b^		Lower CI	Upper CI
			
0–44	6	7.5	0.80	0.29	1.75
45–64	7	10.0	0.70	0.28	1.44
65–74	21	10.1	2.09	1.29	3.19
75+	20	12.7	1.57	0.96	2.42
Total	54	40.4	1.34	1.01	1.75

^a ^Exact 95% confidence intervals calculated for the Poisson distribution^30^.

^b ^Expected numbers based on the NYSDOH Bureau of Cancer Epidemiology Cancer Incidence Standard: 1983–1987, which includes age and sex specific cancer rates for population density Quintile III (suburban). Population of the study area based on 1986 estimated population of the Town of Union based on linear interpolation between 1980 and 1990 Census population estimates.

Spatial analysis techniques have also been used to investigate clustering of leukaemia within central New York [4,5]. Analysis of this well studied data set of 592 leukaemia cases has generally shown an area of increased risk in the Town of Union although the increase has not been statistically significant in all analyses.

The International Agency on Cancer Research (IARC) has classified employment in the boot and shoe manufacturing industry as a Group 1 risk factor, meaning there is sufficient evidence that the exposure or setting is carcinogenic to humans [6]. A number of chemicals used in the shoe and boot manufacturing industry including chlorophenols, hexavalent chromium, aniline and azo dyes and benzene are known or suspected carcinogens. Of these, benzene has most often been implicated as a likely etiologic agent in the development of leukaemia among workers in the industry. IARC has classified benzene exposure as a Group 1 carcinogen [7] and the United States Environmental Protection Agency has also characterized benzene as a known human carcinogen for all routes of exposure based upon convincing evidence from human studies and supporting evidence from animal studies [8,9]. Exposure to benzene has been most strongly associated with acute myeloid leukaemia, the most common type of leukaemia in adults [7,9].

A number of epidemiological reports have shown an association between employment in the shoe and boot manufacturing industry and an increased risk of leukaemia mortality among workers in Italy, Turkey and Great Britain [10-15]. The workers found to be most at risk were those who worked in specific jobs where exposure to solvents and glues containing high levels of benzene was common. In Great Britain, elevated mortality rates were found only among workers in the departments where solvents and glues were used to attach soles to the upper parts of shoes, and exposure to benzene occurred [12]. In Italy there was also evidence that the elevated risk of leukaemia was highest among workers who began work prior to 1963, after which time glues containing high levels of benzene were banned by law [13,14]. A follow-up of the Italian cohort of workers found that the risk of leukaemia increased with increasing cumulative exposure to benzene [15]. Similar results, however, have not been reported in studies of mortality among workers in shoe and boot manufacturing in the United States [16-19].

The Endicott Johnson Company was the major employer in the Town of Union between 1930 and 1960 [20]. The company manufactured shoes and boots in the Endicott and Johnson City area beginning shortly before the turn of the century. At the peak of its activity, in the 1950's, the company employed approximately 20,000 workers in leather tanning and shoe production at numerous factories throughout the Town of Union and neighbouring communities. Beginning in the mid 1960's the company began shifting its manufacturing facilities to areas of the southern United States and eventually overseas. Shoe manufacturing in the Endicott and Johnson City areas continued to decline in 1970's and 1980's as the company focused more on retail sales and eventually ceased production in the mid 1990's.

The objective of the current study is to investigate the association between leukaemia incidence and employment in the Endicott Johnson tanneries and shoe and boot manufacturing facilities, in the Town of Union, Broome County, New York.

## Methods

### Study Population

A matched case-control study was conducted to investigate the association between leukaemia incidence among males 65 and older and employment in the shoe and boot manufacturing industry. The New York State Cancer Registry served as the source for the leukaemia cases. Because the excess of leukaemia was limited primarily to males 65 and older, the selection of cases was restricted to males aged 65 and over with a primary diagnosis of leukaemia (ICD9 204–208) occurring between the years of 1981–1990 and residing in the Town of Union, Broome County, NY at the time of diagnosis. In addition, all cases must have been deceased as of August 1997 and a resident of the Town of Union at the time of death. Since acute myeloid leukaemia (AML) has been most closely associated with exposure to benzene, a subset of the original cases, which included only cases of AML (ICD9 205.0), was also examined. Once the cases were identified from the Cancer Registry, death certificates for each were obtained from the NYSDOH Vital Records Section.

Death certificates for controls were obtained from the NYSDOH Vital Records Section. Four controls meeting the following criteria were selected at random for each case. Controls were restricted to males 65 years and older who resided in the Town of Union at the time of death. In addition, controls were matched to cases on year of death and year of birth +/- 1 year. Matching on dates of birth and death was done to control for potential confounding due to age and to ensure that the cases and controls had a similar opportunity of being exposed (i.e. to work at Endicott Johnson). Additionally, the controls must not have had leukaemia listed as either a cause of death or a contributing cause of death on their death certificates.

Of the 41 leukaemia cases among males 65 and older listed in Table 1, thirty-six cases were identified that met the study criteria; these were matched to 144 controls. Five of the 41 incident cases were eliminated because they were not known to be deceased at the start of the study. For one control the employer could not be identified from any field on the death certificate. A new control was chosen using the predefined restriction and matching criteria. Twelve cases of acute myeloid leukaemia were identified among the 36 leukaemia cases.

### Assessment of Occupation/Exposure

Occupation was determined from information on the death certificate because this represented the most readily available source of occupational information. No company or union records were easily available to determine employment or job status. The company has not allowed previous researches access to company records for occupational health studies [18]. In addition, because of a fairly generous benefits package, labour unions were never able to organize most of the workers under investigation thus union records could not be used as a source of information. [20].

For the purpose of this study an individual was considered exposed if Endicott Johnson was listed on the death certificate under "Name and locality of firm or company". If this field was blank or the employer could not be determined, the fields "usual occupation" and "kind of business" were examined to see if the employer could be determined. The individual responsible for assigning exposure was blinded to case or control status of the study subjects. Because of the ambiguity of many of the listings of occupation on the death certificates, it was not possible to subdivide Endicott Johnson employees further by occupation in order to exclude those employees who had no exposure to carcinogenic chemicals or processes. Therefore all workers in the shoe and boot making factories as well as workers from the nearby Endicott Johnson tanneries were included among the group considered to be exposed.

### Statistical testing

Crude odds ratios (OR) were first calculated for "exposed" versus "non-exposed" groups. However, since each case was individually matched to a set of four controls, the matched sets were analyzed as individual strata. An adjusted OR was therefore calculated taking into account just the contribution of the discordant pairs within each stratum [21]. The SAS Software System V8 was used to calculate the adjusted odds ratios using conditional logistic regression [22]. These data were analyzed separately for all leukaemia cases and their matched controls combined as well as for just cases of AML and their matched controls.

## Results

Approximately 29% of the study subjects (cases and controls) were employed by Endicott Johnson. This was more than the next 5 largest employers in the area combined. Table 2 gives a comparison of the cases and controls according to usual employment at Endicott Johnson. The crude odds ratio for leukaemia among those employed at Endicott Johnson vs. not employed at Endicott Johnson was 1.52 (95%CI 0.70 – 3.30), while the adjusted odds ratio was 1.47 (95%CI 0.70, 3.09). When analysis was restricted to include only those cases of AML, the crude OR was 1.21 (95%CI 0.31, 4.69), while the adjusted odds ratio was 1.19 (95%CI 0.33, 4.28) (Table 3).

**Table 2:** Crude and adjusted risk of leukemia among males 65 and older, by usual employer.

**Employer**	**Cases**	**Controls**	**Total**	**Crude OR (95%CI)**	Adjusted OR (95%CI)*
Endicott Johnson	13 (36%)	39 (27%)	52	1.52 (0.70 – 3.30)	1.47 (0.70 – 3.09)
Other Employer	23 (64%)	105 (73%)	128	**-**	**-**
Total	36	144	180		

*Adjusted Odds Ratio and 95% Confidence Intervals calculated using conditional logistic regression to account for matching on date of birth and age.

**Table 3:** Crude and adjusted risk of AML among males 65 and older, by usual employer.

**Employer**	**Cases**	**Controls**	**Total**	**Crude OR (95%CI)**	Adjusted OR (95%CI)*
Endicott Johnson	4 (33%)	14 (29%)	18	1.21 (0.31 – 4.69)	1.19 (0.33 – 4.28)
Other Employer	8 (67%)	34 (71%)	42	**-**	**-**
Total	12	48	60		

*Adjusted Odds Ratio and 95% Confidence Intervals calculated using conditional logistic regression to account for matching on date of birth and age.

Four study subjects (1 case and 3 controls) had shoe worker as their occupation but their employers were companies other than Endicott Johnson. Odds ratios and confidence intervals were calculated with the subjects considered both as exposed and unexposed. The results of the study were not significantly different. For the results presented here the four subjects were considered as not working for Endicott Johnson (i.e. unexposed). None of these study subjects were part of the AML subgroup.

## Discussion

Overall those whose death certificates indicated that they worked at Endicott Johnson had approximately a 50% higher risk of developing leukaemia than those who did not work for the company; however, there was not enough evidence to rule out the possibility that the observed results were due to chance alone. Among the types of leukaemia, occupational exposures have been most closely associated with acute myeloid leukaemia; thus we might have expected to observe a greater risk of AML among Endicott Johnson workers. This was not the case, however, as the risk of AML was slightly less than that observed for all leukaemias.

Previous studies of workers employed in the shoe and boot manufacturing industry in the United States have failed to find an elevated risk for leukaemia mortality [16-19]. In contrast, several occupational studies in Italy and Great Britain have reported an elevated risk of leukaemia mortality among shoe and boot workers [12-15]. While we did observe an elevated risk of leukaemia among workers in the current study, the excess was not statistically significant. Differences in study design may have accounted for some of the variation in results observed between the European and US studies. The British and Italian studies were retrospective cohort studies, whereas three of the four US studies were proportional mortality studies. The use of a cohort study design allows better exposure assessment and control for confounding. The only US cohort study was of shoe workers in a factory where benzene was never used as a solvent in glues as it had been in the European factories [19]. Although the current study used a case control study design, many of the limitations of the proportional mortality studies also existed in the current study.

Limitations that may have prevented the observance of more conclusive findings including small sample size, limited work history, no exposure assessment and limited control of confounding factors. Table 4 describes several potential sources of bias in the study and how they may have affected the results.

**Table 4:** Potential forms of bias and the direction that they may have influenced the results.

**Cause or Source of Bias**	**Direction of the Effect**
Accuracy of "Usual Occupation" on Death Certificate	Either (most likely toward the null)
Only deceased individuals eligible as controls	Toward the null
Lack of specific job titles	Toward the null
Migration into and out of region	Toward the null
No information on length of employment	Toward the null
No information on specific chemicals used	Toward the null

The power to detect a true increase in risk was limited by the population size of the study. Because this study was a follow-up to a previous study, the population was limited by the constraints of the previous study. Leukaemia is a relatively rare disease and the population of white males over 65 in the study area was only approximately 3,500. For the current study the power to detect an increased risk of 50% (similar to the observed risk for leukaemia) with a 95% confidence interval was only 20%. In order to achieve a power of 80% the study population would have had to have been approximately eight times as large. This could have been achieved by either increasing the study area to include neighbouring communities or by increasing the period under observation. However, as mentioned previously we were limited by the parameters of the previous study.

The accuracy of occupation and industry data found on death certificates has been examined in a number of studies [23-27] and their value in epidemiological studies has been questioned by some [26,28]. Studies have generally found industry listed on the death certificate to be accurate between 50% and 80% of the time when compared to data collected from the next of kin or from company records. The accuracy of occupation/industry information of death certificates used in the current study, however, was probably in the higher end of those ranges for several reasons. The current study was limited to white males and previous studies on the accuracy of industry and occupation on death certificates have found a higher concordance with other data sources among whites compared to blacks [26]. Since we were seeking information on employer and industry not on specific occupations, we also believe this lead to greater accuracy. Concordance between "company" found on death certificates compared to work histories has been found to be accurate 81% of the time while among those retired at the time of death 91% concordance has been reported [27]. In addition, because Endicott Johnson was the major employer in the area at the time, it was more likely to be known by the next of kin. Nonetheless it is likely there was some misclassification of employer on the death certificates. Usual occupation as listed on the death certificate may not have been accurate for workers who switched jobs late in life. However, this type of misclassification has been found to occur in a random manner, thus, it would have likely biased the results slightly toward the null [25].

In addition, only dead individuals were eligible as controls in the study. Since working in the shoe and boot manufacturing industry is associated with other kinds of types of cancer, such as lung and bladder cancer, in addition to leukaemia, this may have artificially increased the prevalence among controls. This would also bias the odds ratio towards the null.

A major limitation in this study was the assessment of exposure. Because death certificates were used to identify usual employer and occupation, information on specific jobs worked was fairly limited and often missing completely. Even when the employer was known to be Endicott Johnson, a nondescript job title such as "shoe worker", "Endicott Johnson worker", or "labourer" was often given for occupation. Because of this, any mention on the death certificate of having worked at Endicott Johnson was used as a surrogate for exposure. The effect of including every Endicott Johnson worker in the exposed group may have biased the odds ratio toward the null due to the large variety of jobs within each factory. In addition to all workers in the shoe and boot making factories, workers from the nearby Endicott Johnson tanneries were also included among the group considered to be exposed, further diluting the effect. Previous studies have found an increase in leukaemia mortality only among men in departments where shoes were assembled [12-15].

Additionally, it was not known what chemicals were used in the production of shoes at the Endicott Johnson facilities. Therefore, it is difficult to know if the lack of an association between leukaemia and employment for Endicott Johnson may have been a result of poor exposure assessment or a result of no exposure to certain carcinogens.

The length of employment and timing of the exposure were also not taken into account. However, the "usual occupation" as listed on the death certificate is most likely to represent long-term, stable employment. In Italy, the highest leukaemia rates were found among workers who worked in the industry prior to 1963 [13]. Estimates for the latency between occupational exposure to carcinogens and the development of leukaemia range from 2 to 20 years. Therefore, the temporality of the exposure and disease development suggests that many of the cases would have occurred before our study period. In addition, previous studies have found that the biggest increase in leukaemia mortality occurred among male workers less than 50 [9]. In the current study we focused exclusively on males over 65 because rates among males less than 50 were not significantly elevated.

Migration is a problem when investigating diseases such as cancer that have a long latency period. In the current study this may have been compounded by the fact that the workforce at Endicott Johnson has been declining since the mid 1950's as the company slowly scaled back and eventually eliminated its production facilities in the area. In addition, both cases and controls may have retired and moved out of the area. Overall, however, it is felt that those males age 65 and older in this region represent a fairly stable population. Nonetheless, any misclassification due to migration would certainly bias the results toward the null since it is unlikely that any cases who recently moved into the area would have worked for Endicott Johnson.

Most types of bias identified in this study would tend to lead to an underestimation of the true risk (i.e. bias the results toward the null). A summary of the types of biases identified and their effects is given in Table 4. Because most would lead to an underestimation of risk it is likely that the true association between working for Endicott Johnson and the risk of leukaemia is somewhat higher than indicated. However to fully evaluate these biases a more thorough study design is needed.

In the study only age, gender and date of birth were controlled. Because of the study design, other possible confounders could not be taken into account. For instance, cigarette smoking has been associated with several forms of leukaemia, however no attempt was made to determine smoking status in the current study [29].

## Conclusions

The incidence of leukaemia was of interest in this community because a number of previous cancer investigations in the area have found an increase in leukaemia incidence. In addition, several studies have found an association between leukaemia and employment in the shoe and boot manufacturing industry, which was predominant in the town for most of the past century. In the current study, a positive association between the risk of leukaemia and working at Endicott Johnson was observed; however, the small population size prevented us from determining whether the results could be due to chance alone. Serious limitations may have prevented the observance of more conclusive findings in this study. Better exposure assessment, information on length of exposure and types of job held, control of confounding factors and information on chemicals used by this company would strengthen any future investigations. However, many of the most carcinogenic chemicals, which at one time were used in the industry, have not been used for several decades. In addition, since the company no longer manufactures shoes and boots, there is no current or future exposure among this particular group. Nonetheless, lessons learned from retrospective analyses of disease among workers in American industries may be applicable to overseas industries, particularly in developing nations, where many of the safeguards and restrictions that have been in place for decades in the US and Europe have not yet been adopted.

## List of Abbreviations

AML, acute myeloid leukaemia

CI, confidence interval

IARC, International Agency on Cancer Research

ICD, International Classification of Diseases

NYSDOH, New York State Department of Health

OR, odds ratio

## Competing interests

None declared.

## Authors' contributions

Not applicable.
